# A Narrative Review: Relationship Between Glycemic Variability and Emerging Complications of Diabetes Mellitus

**DOI:** 10.3390/biom15020188

**Published:** 2025-01-28

**Authors:** Xinxin Wang, Yanli Cao

**Affiliations:** Department of Endocrinology and Metabolism, Institute of Endocrinology, NHC Key Laboratory of Diagnosis and Treatment of Thyroid Diseases, The First Affiliated Hospital of China Medical University, Shenyang 110001, China; xxwang2024@cmu.edu.cn

**Keywords:** glycemic variability, oxidative stress, diabetes, emerging complications

## Abstract

A growing body of evidence emphasizes the role of glycemic variability (GV) in the development of conventional diabetes-related complications. Furthermore, advancements in diabetes management and increased life expectancy have led to the emergence of new complications, such as cancer, liver disease, fractures, infections, and cognitive dysfunction. GV is considered to exacerbate oxidative stress and inflammation, acting as a major mechanism underlying these complications. However, few reviews have synthesized the association between GV and these emerging complications or examined their underlying mechanisms. Hence, this narrative review provides a comprehensive discussion of the burden, risks, and mechanisms of GV in these complications, offering further evidence supporting GV as a potential therapeutic target for diabetes management.

## 1. Introduction

Diabetes mellitus (DM) is a major health issue, and its prevalence is steadily increasing globally. Over half a billion people worldwide are estimated to be affected by this disease [1]. The conventional complications of DM, particularly vascular issues, continue to burden millions of individuals. Moreover, advances in DM management strategies and increased life expectancy have led to the emergence of a distinct set of less-recognized complications, as supported by a growing body of evidence [2]. DM is linked to an increased risk of cancer, neurodegenerative diseases, cognitive disorders, liver disease, functional disability, affective disorders, sleep disturbance, and infections [3,4,5,6,7,8,9]. In some regions, cancer and dementia have been reported as the leading causes of death in patients with DM [10,11]. Although conventional complications continue to constitute the majority, their incidence and mortality rates are declining annually, probably giving way to emerging complications [12].

Glycemic variability (GV) refers to fluctuations in blood glucose levels and plays a key role in maintaining glucose homeostasis [13,14]. It is typically defined by measuring glucose levels or other parameters related to glucose homeostasis over specified time intervals. Based on the duration of these intervals, GV can be categorized into short-term and long-term variability. Short-term GV is commonly assessed using metrics such as the standard deviation (SD), coefficient of variation (CV), and mean amplitude of glycemic excursions (MAGE), with MAGE often regarded as the “gold standard” for evaluating within-day fluctuations. Long-term GV is measured through periodic assessments, such as hemoglobin A1c (HbA1c), fasting plasma glucose (FPG), and postprandial glucose, with SD and CV commonly used to quantify long-term glucose fluctuations [14,15]. A substantial body of evidence has indicated that both short-term and long-term GVs increase the risk of DM-related complications and associated mortality [15,16,17,18]. GV is a clinically relevant and valuable parameter, as it can be measured easily, independent of the prandial status, and is potentially modifiable. Despite considerable evidence supporting the role of GV in the progression of diabetic complications, few articles have summarized and discussed the link between GV and emerging complications.

This review consolidates the current knowledge on the role of GV in the onset and progression of emerging DM-related complications, including cancer, liver disease, bone disease, functional disability, neuropsychiatric disorders, and infections, while exploring its underlying mechanisms and impacts.

## 2. Materials and Methods

We conducted a literature search of PubMed to identify English-language articles published from the database’s inception through November 30, 2024. The search terms included “glycaemic variability”, “glycemic variability”, “glucose variability”, “fasting glucose variability”, “HbA1c variability”, “glucose fluctuation”, and “oscillating glucose.” Our review focused on studies examining the relationship between glycemic variability and the emerging complications of DM. We prioritized studies published from 2020 onward, all of which were in English. Additionally, relevant older references were included where appropriate to ensure comprehensive coverage of the topic.

## 3. GV and Cancer

### 3.1. Relationship Between GV and Cancer in Clinical Studies

DM is associated with a heightened lifetime risk of developing malignancies [19]. However, the underlying biological links between these two diseases remain poorly understood. GV effectively reflects the level of blood glucose control, and its role in cancer development is being increasingly recognized.

GV may be a strong predictor of cancer incidence and mortality in patients with DM. A study performed in 2000 reported that GV is a strong predictor of cancer mortality in elderly patients with type 2 diabetes mellitus (T2DM), specifically in those aged 56–74 years, who constitute the majority of the diabetic population [20]. Another study documented that compared with the first tertile, the hazard ratios for cancer incidence and mortality in the third tertile of annual FPG-CV were 3.03 (95% CI: 1.98–4.65) and 5.04 (95% CI: 2.32–10.94) [21] (Table 1). Interestingly, another study found that visit-to-visit HbA1c variability is a potential risk factor for subsequent tumor development and displays a dose-dependent association with tumor occurrence. This relationship is especially evident when comparing groups containing a high GV with those having the lowest GV [22]. Two retrospective cohort studies conducted in Shanghai, China, demonstrated a positive association between long-term fluctuations in fasting blood glucose (FBG) levels and cancer risk in patients with T2DM [23,24]. Interestingly, this study revealed a significant negative interaction effect between FBG variability and hypertension on cancer risk, whereas the mean FBG level did not exert a significant effect. Two potential explanations for this finding are as follows: the results could have been influenced by confounding factors, or the coexistence of hypertension and FBG variability could have modulated the individual effects of each factor despite their independent contributions to cancer development [24].

Subsequent studies have also examined the association between GV and site-specific cancers. In the prospective Hong Kong Diabetes Register cohort (1995–2019), increased GV was linked to an elevated risk of all-cause mortality, as well as deaths from breast, liver, and cancer-specific causes in individuals with T2DM [17]. Notably, GV appears to affect even individuals without DM. A study found that high visit-to-visit variability in FPG levels in a DM-free population was independently linked to an elevated risk of gastric and colorectal cancers [25,26].

In contrast, a 2012 study involving 754 patients with T2DM observed that following a 2-year follow-up, mean HbA1c, rather than HbA1c variability, was predictive of cancer mortality. The researchers attributed the observed association of HbA1c variability with all-cause mortality to the insufficient strength of the link with cancer-related mortality compared with its stronger correlation with noncancer mortality, including cardiovascular disease [27]. Overall, GV may play a significant role in clinical practice for care management and cancer prevention [21]. Routine cancer screening should be considered for patients with DM and unstable glycemic control [22].

**Table 1:** A summary of original studies on cancer risk associated with GV.

Study	DM Status and Type	GV: Method of Assessment and Parameters	Study Type Included (n)	Outcome	Risk Associated with GV (95% Confidence Interval)	
Lin et al. (2012) [21]	T2DM	Visit-to-visit variability of FPG	Cohort (4805)	Cancer	Group (FPG-CV, %)	HR
					≤14.41	Reference
					14.41–25.10	1.66 (1.01, 2.71) *
					>25.10	3.03 (1.98, 4.65) *
Saito et al. (2019) [22]	Any DM	Visit-to-visit variability of HbA1c	Cohort (2640)	Cancer	Group (HbA1c, mean (SD), %)	OR
					6.66 (0.96)	Reference
					6.69 (0.71)	1.20 (0.88–1.65)
					6.98 (0.98)	1.43 (1.02–2.00) *
					7.78 (1.51)	2.19 (1.52–3.17) *
Yoo et al. (2021) [28]	Any DM	Visit-to-visit variability of FPG	Cohort (674,178)	Hepatocellular carcinoma	Group (Quartile of CV)	HR
					Q1	Reference
					Q2	1.05 (0.97–1.13) *
					Q3	1.09 (1.01–1.18) *
					Q4	1.23 (1.14–1.33) *
Mao et al. (2022) [17]	Any DM	Visit-to-visit variability of HbA1c	Cohort (15,286)	Cancer	HR 1.13 (1.03–1.24) *	
				Breast cancer	HR 1.30 (0.97–1.75)	
				Liver cancer	HR 1.37 (1.09–1.74) *	
				Colorectal cancer	HR 1.08 (0.90–1.30)	
Jun et al. (2022) [26]	No DM	Visit-to-visit variability of FBG	Cohort (246,241)	Cancer	Group (Quintiles of SD)	HR
					Q1: <4.97	Reference
					Q2: 4.97–7.49	0.98 (0.88–1.10)
					Q3: 7.50–10.11	1.16 (1.04–1.28) *
					Q4: 10.12–14.19	1.10 (0.99–1.22)
					Q5: ≥14.20	1.32 (1.19–1.46) *
Cui et al. (2022) [24]	T2DM	Visit-to-visit variability of FBG	Cohort (46,761)	Cancer	Group	HR
					Normotension	1.38 (1.13–1.68) *
					Hypertension	1.02 (0.92–1.13)

HR, hazard ratio; OR, odds ratio; GV, glycemic variability; DM, diabetes mellitus; T2DM, type 2 diabetes mellitus; Any DM, any type of diabetes; No DM, no diabetes; FPG, fasting plasma glucose; FBG, fasting blood glucose; HbA1c, hemoglobin A1c; CV, coefficient of variation; SD, standard deviation; * *p* < 0.05, statistically significant.

### 3.2. Potential Mechanisms of GV in Cancer

GV activates the release of inflammatory cytokines, promotes the adhesion of monocytes and macrophages, and increases oxidative stress, thereby initiating a cascade of pathological events. These processes collectively result in endothelial dysfunction and generalized vasculopathy [29,30,31,32]. This adverse metabolic environment inhibits apoptosis and facilitates the proliferation of neoplastic cells, disrupting cellular homeostasis [17].

Oxidative stress, primarily driven by mitochondrial reactive oxygen species (ROS), plays a pivotal role in tumorigenesis. This stress influences cell proliferation, migration, invasion, angiogenesis, inflammation, immune evasion, and other cancer-related processes. Paradoxically, oxidative stress-induced cellular senescence may serve as a protective mechanism, preventing the transformation of normal cells into malignant cells [33,34]. Chronic inflammation, often associated with oxidative stress, exacerbates the risk of cancers such as gastric, colorectal, and hepatic malignancies. Tumors themselves induce inflammatory responses, aggravating endothelial dysfunction [35,36]. Recent studies have established a significant association between microvascular endothelial dysfunction and an elevated risk of solid tumors [37,38].

Enhanced GV has been implicated in increased platelet reactivity, a process intricately associated with cancer progression via platelet activation and coagulation pathways [39,40]. Platelets play a crucial role in enhancing tumor growth, metastasis, immune evasion, and tumor angiogenesis [41]. Activated platelets release growth factors that support tumor proliferation, angiogenesis, and metastasis [42]. Moreover, cancer cells can activate platelets, using them as protective shields against blood shear forces and natural killer cells [43]. This interaction creates a reciprocating cycle in which tumor-induced platelet activation stimulates tumor growth and dissemination, simultaneously triggering thrombosis and amplifying tumor progression.

Recent studies have identified a network of 37 genes and proteins related to GV that influence glucose metabolism, signaling, and cell proliferation. These genes and proteins play central roles in pathways linked to diabetic vascular complications [44]. Prioritization analysis highlighted genes such as fibronectin 1 (*FN1*) and thrombospondin 1 (*THBS1*). *FN1* is associated with tumor relapse and poor prognosis in patients with gastric cancer and leads to cisplatin resistance, proliferation, migration, invasion, and reduced apoptosis in cervical cancer cells [45,46]. *THBS1* is linked to immunosuppression and unfavorable outcomes in colorectal cancer [47]. In addition, collagen type VI alpha 1 (ChainCOL6A1), regulated by GV, is correlated with poor prognosis in patients with pancreatic cancer, which highlights the role of GV in cancer progression [48].

## 4. GV and Liver Diseases

### 4.1. Relationship Between GV and Liver Diseases in Clinical Studies

Several studies have explored the association between dysglycemia and the risk of metabolic dysfunction-associated steatotic liver disease (MASLD). A Chinese cohort study involving 512,891 adults who were followed up for 10 years found that DM was linked to an adjusted HR of 1.76 (95% CI: 1.47–2.16) for MASLD compared with nondiabetic individuals [49]. Furthermore, a study has asserted the presence of a risk gradient for MASLD, even within the nondiabetic glycemic range. A large cross-sectional study of 99,969 healthy, nondiabetic individuals categorized HbA1c levels into four groups (≤4.9%, 5.0–5.4%, 5.5–5.9%, and 6.0–6.4%). Compared with the lowest quartile (HbA1c ≤4.9%), higher HbA1c levels were progressively linked to increased MASLD risk, with ORs of 1.44, 2.62, and 7.18, respectively [50]. These findings emphasize the role of dysglycemia in the development and progression of MASLD.

GV is increasingly recognized as a crucial indicator of glycemic control [15], which may play a pertinent role in the development of MASLD. This prospective cohort study included 2467 adults aged 18–30 years who were followed up for 25 years. This investigation noted that greater visit-to-visit FPG variability in early adulthood was related to a higher risk of MASLD in middle-aged individuals, independent of mean FPG levels [51] (Table 2). Similarly, in a longitudinal cohort study of 21,123 participants who were followed up for a median of 57 months, high HbA1c variability was significantly linked to incident MASLD in the DM group (adjusted OR 1.14, 95% CI 1.01–1.29) but not in the normal glucose tolerance or preDM group [52]. However, GV may also be crucial in nondiabetic individuals. A study of 57,636 Korean adults without MASLD or DM reported that greater long-term FPG variability was independently associated with the development of MASLD. This correlation was more pronounced in individuals with a normal body mass index (BMI) [53]. A study involving 200 individuals with T1DM observed that the controlled attenuation parameter, an indicator of hepatic steatosis, was positively correlated with CV exclusively in lean individuals (BMI ≤25 kg/m^2^. This observation suggests an association between blood glucose variability and hepatic steatosis in the absence of overweight or obesity [54]. This result could be attributed to the diminishing effect of GV on MASLD as body weight increases. Obesity and higher adiposity worsen insulin resistance and chronic inflammation, both of which are critical factors in MASLD pathogenesis. A study of 169 patients with biopsy-confirmed MASLD documented that the median glucose levels were significantly higher in patients with severe fibrosis than in those with mild fibrosis. This finding implies that GV is an independent predictive factor for the progression of hepatic fibrosis in MASLD [55].

The link between MASLD and dysglycemia, as represented by GV, appears to be bidirectional. MASLD was independently linked to an increased risk of severe hypoglycemia in individuals with T2DM, regardless of their obesity status. Therefore, the presence of MASLD should be considered when evaluating hypoglycemia risk in patients with T2DM [56].

### 4.2. Potential Mechanisms of GV in Liver Diseases

MASLD involves complex pathogenesis and typically begins with hepatic steatosis, driven by factors such as insulin resistance and metabolic abnormalities. Fat accumulation in the liver progressively worsens oxidative stress, chronic inflammation, and lipid metabolism disturbances, leading to hepatocyte damage and immune response activation. These processes promote liver inflammation and fibrosis, which can eventually progress to cirrhosis and even liver cancer [57].

GV induces cellular metabolic memory via epigenetic mechanisms, such as chromatin remodeling and nonenzymatic glycosylation. Chronic inflammation and oxidative stress are thus maintained, leading to abnormal metabolic states in the long term. These metabolic impairments may persist even if blood glucose levels improve, exerting lasting effects on the liver [58,59,60,61]. Furthermore, GV contributes to insulin resistance by disrupting lipid metabolism in the liver. Insulin resistance enhances lipolysis and increases fatty acid delivery to the liver while simultaneously inhibiting β-oxidation. These processes promote triglyceride accumulation in the liver [62,63,64,65].

In addition, investigations have documented that blood GV has a more pronounced effect on oxidative stress and inflammatory factors than on sustained hyperglycemia [29,32]. This fluctuation may trigger monocyte adhesion to endothelial cells, activating inflammatory cytokines, and exacerbating liver inflammation [55]. Moreover, increased oxidative stress levels may impair mitochondrial function, accelerating hepatocyte apoptosis, inflammation, and fibrosis in a lipotoxic environment [66]. These events ultimately result in the progression of MASLD.

## 5. GV and Bone Disease, Functional Disability

### 5.1. Relationship Between GV and Bone Disease, Functional Disability in Clinical Studies

Skeletal fragility is a common complication of DM; however, it does not always correlate with low bone mass or trauma severity in patients with DM. Unlike osteoporotic bone disease, which is characterized by decreased bone mass, both T1DM and T2DM are associated with impaired bone remodeling and turnover, which compromise bone material quality [67]. GV may affect bone metabolism via multiple pathways, resulting in an imbalance between bone resorption and bone formation. GV itself may induce high mineralization, disrupt bone metabolism, and aggravate the risk of fractures. The amplitude of GV and elevated dawn glucose levels have been reported to be negatively correlated with bone turnover markers [68]. Furthermore, high variabilities in body weight and blood glucose levels are linked to an increased incidence and risk of hip fractures in patients with DM [69] (Table 3). HbA1c variability is an independent predictor of increased hip fracture risk in individuals with T2DM, irrespective of the level of glycemic control. Therefore, from a bone health perspective, maintaining elevated HbA1c levels is not recommended [70].

Both Korean and Chinese studies support the conclusion that high FPG variability is a risk factor for osteoporotic fractures in the nondiabetic population. Previous research established that individuals in the highest quartile of FPG variability showed an 11% increased risk of total fractures and a 16% elevated risk of vertebral fractures compared to those in the lowest quartile [71]. The latter study demonstrated a 32% higher risk of osteoporotic fractures in the third tertile of FPG variability compared with the first tertile in the nondiabetic population. In addition, increased FPG variability was associated with a higher risk of osteoporotic fractures in patients with DM (47%) than in those without DM (32%) [72].

Disability is defined as difficulty in functioning in one or more life domains, as experienced by an individual with a health condition in interaction with contextual factors [73]. Data from a population-based sample of U.S. adults indicated a link between visit-to-visit HbA1c variability and the number of physical functioning difficulties, independent of mean HbA1c levels, in individuals aged more than 50 years [74].

Moreover, an observational study based on a large nationwide cohort in Taiwan identified visit-to-visit GV as a significant predictor of minor lower extremity amputation (LEA) risk. Even after adjusting for conventional risk factors, this association remained robust. The effect of GV was particularly pronounced in patients with a longer duration of DM (>3 years) and normal kidney function (eGFR ≥60 mL/min/1.73 m^2^). These observations emphasize that managing GV could play a crucial role in alleviating the risk of LEA [75].

**Table 3:** A summary of original studies on bone diseases and functional disability risks associated with GV.

Study	DM Status and Type	GV: Method of Assessment and Parameters	Study Type Included (n)	Outcome	Risk Associated with GV (95% Confidence Interval)	
Lee et al. (2022) [69]	Any DM	Visit-to-visit variability of FPG	Cohort (480,539)	Hip fracture	Group (Quartiles of VIM)	HR
					Q1:	Reference
					Q2:	0.97 (0.87–1.08)
					Q3:	1.20 (1.08–1.33) *
					Q4:	1.34 (1.21–1.48) *
Lui et al. (2020) [70]	T2DM	Visit-to-visit variability of HbA1c	Cohort (83,282)	Hip fracture	Group (HbA1c-CV)	HR
					Q1: <4.78	Reference
					Q2: 4.78–7.47	1.08 (0.97–1.20)
					Q3: 7.48–11.49	1.17 (1.06–1.31) *
					Q4: ≥11.50	1.46 (1.32–1.62) *
Kim et al. (2021) [71]	No DM	Visit-to-visit variability of FPG	Cohort (92,929)		Group (Quartiles of FPG-SD)	HR
				Total fracture	Q1:	Reference
					Q2:	1.00 (0.93–1.08)
					Q3:	0.94 (0.87–1.01)
					Q4:	1.11 (1.03–1.20) *
				Vertebral fracture	Q1:	Reference
					Q2:	0.92 (0.80–1.05)
					Q3:	1.01 (0.88–1.15)
					Q4:	1.16 (1.02–1.31) *
Liu et al. (2023) [72]	ALL	Visit-to-visit variability of FPG	Cohort (57,295)	Osteoporotic fracture	Group (FPG-SD)	HR
					T1: <0.33	Reference
					T2: 0.33–0.60	1.07(0.89–1.29)
					T3: ≥0.60	1.32(1.10–1.60) *
Shao et al. (2022) [74]	ALL	Visit-to-visit variability of HbA1c	Cohort (5544)	Functional limitation	Group (FPG-CV)	OR
					mean HbA1c	0.98 (0.09–1.02)
					HbA1c CV	1.88 (1.14–3.10) *
Li et al. (2020) [75]	T2DM	Visit-to-visit variability of FPG, HbA1c	Cohort (27,574) DM duration >3 years	Lower extremity amputation	Group (FPG-CV%)	HR
					<17.5	Reference
					17.5–34.7	1.53 (1.07–2.18) *
					≥34.8	1.94 (1.38–2.72) *
					Group (HbA1c-CV%)	HR
					<8.4	Reference
					8.4–16.6	1.18 (0.86–1.60)
					≥16.7	1.51 (1.12–2.05) *

HR, hazard ratio; OR, odds ratio; GV, glycemic variability; DM, diabetes mellitus; VIM, variability independent of the mean; T2DM, type 2 diabetes mellitus; Any DM, any type of diabetes; No DM, no diabetes; ALL, with or without diabetes; FPG, fasting plasma glucose; HbA1c, hemoglobin A1c; CV, coefficient of variation; SD, standard deviation; * *p* < 0.05, statistically significant.

### 5.2. Potential Mechanisms of GV in Bone Disease and Functional Disability

Oxidative stress resulting from GV contributes to endothelial dysfunction, activation of calcifying vascular cells, and osteoblast dedifferentiation. These processes accelerate bone remodeling and augment bone loss [29,76,77].

When glucose and insulin metabolism is disrupted, osteoblast and osteoclast activities are directly affected, thereby reducing bone formation and resorption. These changes, in turn, decrease bone remodeling and bone mass and increase fracture risk [67]. Chronic systemic inflammation with an increase in insulin resistance influences bone strength and mass, aggravating the risk of fractures [78,79]. Furthermore, the “bone–vascular axis” describes the interrelationship among the vascular system, bone-derived cells, and endocrine hormones, significantly affecting vascular health [80,81]. Disruptions in glucose and insulin metabolism damage the bone vasculature, impairing the delivery of nutrients and signaling factors (such as those regulating vascular dilation) to the bone tissue. This disruption is particularly prominent in DM, where alterations in the bone vasculature result in reduced bone remodeling activity [67]. Damage to the microvascular system is closely associated with the deterioration of bone microstructure, and high FPG-GV may further decrease bone quality and increase fracture risk [81,82]. These results highlight the essential role of the interplay between metabolic and vascular health in sustaining skeletal integrity.

Intracellular hyperglycemia results in overproduction of superoxide at the mitochondrial level, which escalates the production of advanced glycation end products (AGEs) [83]. The accumulation of these products induces pathways that stimulate collagen crosslinking, alter bone biomechanics, and affect bone remodeling [67]. Chronic hyperglycemia induces glycation and oxidation of type I collagen, converting enzymatic crosslinks into nonenzymatic AGEs, which decreases bone material strength and triggers osteoblast apoptosis. In addition, enhanced sclerostin production by osteocytes inhibits the WNT signaling pathway, suppressing bone turnover [84,85].

Individuals with greater blood glucose variability are more prone to neuropathy and ischemia, which can exacerbate the risk of ulcers and infections, potentially leading to amputation [75]. Furthermore, blood glucose variability is linked to decreased muscle strength and mass and accelerated muscle loss, which negatively impacts fracture prognosis and is associated with functional disability [74]. These combined mechanisms lead to bone disease and functional disability in patients with conditions, such as DM.

## 6. GV and Neuropsychiatric Disorders

### 6.1. Relationship Between GV and Neuropsychiatric Disorders in Clinical Studies

Patients with DM are vulnerable to dementia and cognitive impairment, which makes comprehensive self-care management difficult [86]. Therefore, modifiable factors related to dementia must be identified. Recent research has shown a strong association between GV and neuropsychiatric disorders, emphasizing the importance of stable glycemic control. A study involving 837 Israeli participants reported that each 1% increase in HbA1c-SD corresponded to a 1.31-fold increase in depressive symptoms, suggesting that GV reduction may mitigate depression risk in elderly individuals with T2DM [87] (Table 4). Late-life depression, which is closely associated with cognitive impairment, is hypothesized to share common underlying mechanisms with cognitive decline [88].

Evidence from two large cohorts (HRS and ELSA) with a mean follow-up of 10.48 years revealed that greater HbA1c variability was linked to accelerated cognitive decline in nondiabetic individuals independent of average HbA1c levels. Nevertheless, this relationship was not observed in individuals with diabetes [89]. Another study demonstrated significant sex-based differences in the effect of glycemia-related risk factors on Alzheimer’s disease (AD) in T2DM. Severe hypoglycemia and HbA1c variability were found to independently predict AD incidence in women, but not in men, over a period of 6 years. These findings highlight the dire need for glycemic stability, particularly in older women [90].

In a Korean cohort study with a 6.9-year follow-up period, higher fasting glucose variability was linked to an elevated risk of all-cause dementia (18%), AD (19%), and vascular dementia (17%). This association was particularly evident in nonobese individuals with prolonged DM, dyslipidemia, and glucose-lowering treatments. These results confirm that fasting glucose variability is a cost-effective target for public health interventions to alleviate dementia risk [91]. Moreover, GV measured as variability independent of the mean (VIM) exhibited a significant negative correlation with cognitive function scores on the Mini-Mental State Examination (r = −0.729) and Montreal Cognitive Assessment (r = −0.710), establishing VIM as an independent risk factor for cognitive impairment [92].

In addition, patients in the highest quartile of glucose variability demonstrated a significantly elevated risk of Parkinson’s disease dementia than those in the lowest quartile (SHR = 1.50, 95% CI 1.19–1.88) [93]. Moreover, a large GV was linked to slower and less accurate processing, although slight glucose elevations relative to individual means were linked to faster processing, further asserting the cognitive impact of GV [94]. These findings establish the importance of achieving stable glycemic control to preserve cognitive and mental health.

**Table 4:** A summary of original studies on neuropsychiatric disorder risk associated with GV.

Study	DM Status and Type	GV: Method of Assessment and Parameters	Study Type Included (n)	Outcome	Risk Associated with GV (95% Confidence Interval)	
Ravona-Springer et al. (2017) [87]	T2DM	Visit-to-visit variability of HbA1c	Cohort (837)	Depression	Group	IRR
					-	1.31 (1.03–1.67) *
Lee et al. (2021) [90]	T2DM	Visit-to-visit variability of HbA1c	Cohort (85,514)	Alzheimer’s disease	Group (Women)	HR
					Severe hypoglycemia	1.69 (1.14–2.52) *
					Adjusted SD	1.15 (1.02–1.30) *
Lee et al. (2022) [91]	Any DM	Visit-to-visit variability of FPG	Cohort (769,554)		Group (VIM)	HR
				All-cause dementia	Q1: 0–12.7	Reference
					Q2: 12.8–20.5	1 (0.98–1.03)
					Q3: 20.6–31.2	1.07 (1.04–1.09) *
					Q4: ≥31.3	1.18 (1.15–1.21) *
				Alzheimer’s disease	Q1: 0–12.7	Reference
					Q2: 12.8–20.5	1.01 (0.98–1.04)
					Q3: 20.6–31.2	1.08 (1.05–1.11) *
					Q4: ≥31.3	1.19 (1.15–1.22) *
				Vascular dementia	Q1: 0–12.7	Reference
					Q2: 12.8–20.5	0.98 (0.91–1.04)
					Q3: 20.6–31.2	1.06 (0.99–1.13)
					Q4: ≥31.3	1.17 (1.09–1.25) *
Kang et al. (2023) [93]	ALL	Visit-to-visit variability of FPG	Cohort (9264)	Parkinson’s disease dementia	Group (Quartiles of FPG-CV)	SHR
					Q1:	Reference
					Q2:	1.30 (1.04–1.63) *
					Q3:	1.29 (1.02–1.62) *
					Q4:	1.50 (1.19–1.88) *
	No DM				Q1:	Reference
					Q2:	1.18 (0.96–1.45)
					Q3:	1.20 (0.97–1.49)
					Q4:	1.46 (1.17–1.83) *

HR, hazard ratio; IRR, incidence rate ratios; SHR, subdistributional hazard ratio; VIM, variability independent of the mean; GV, glycemic variability; DM, diabetes mellitus; T2DM, type 2 diabetes mellitus; Any DM, any type of diabetes; No DM, no diabetes; ALL, with or without diabetes; FPG, fasting plasma glucose; HbA1c, hemoglobin A1c; CV, coefficient of variation; SD, standard deviation; * *p* < 0.05, statistically significant.

### 6.2. Potential Mechanisms of GV in Neuropsychiatric Disorders

GV is a pertinent factor in the onset and progression of neuropsychiatric disorders, such as cognitive decline, dementia, and depression. Oxidative stress and inflammation are key mechanisms by which GV amplifies reactive ROS, leading to neuronal damage and endothelial dysfunction [95]. This oxidative environment facilitates the formation of AGEs, accelerating amyloid-beta deposition and tau protein hyperphosphorylation, which are hallmarks of AD [96,97].

GV-induced insulin resistance is another pivotal pathway that is involved in neurodegeneration. GV results in excessive insulin secretion, leading to peripheral and cerebral insulin resistance. This disrupts insulin signaling, reduces cerebral perfusion, and increases neuronal vulnerability [98,99]. GV also exacerbates microvascular complications, enhancing cerebral small vessel damage, which is linked to cognitive impairment and vascular depression [100,101].

Mitochondrial dysfunction—arising from GV-induced disruption of the mitochondrial respiratory chain—heightens the susceptibility of neurons and pancreatic beta cells, exacerbating neurodegeneration [102,103,104]. Furthermore, changes in protein levels, such as increased ApoA1 and decreased C3, are associated with a reduced risk of AD, suggesting that tight glucose control may help mitigate cognitive decline [105,106,107]. Hypoglycemic events, especially in elderly patients, can worsen neurotoxic effects [108,109]. Women are particularly vulnerable to the adverse cognitive effects of GV, which could be attributed to sex-specific genetic and hormonal interactions, such as those involving ApoE4 genotypes [110,111]. Therefore, these APoE genotype–phenotype interactions may be involved in the sexual dimorphism observed in the link between GV and AD risk.

Effective strategies to control GV and mitigate its downstream effects are essential to reduce the burden of neuropsychiatric disorders in vulnerable populations. Strict blood glucose control may not only help slow the progression of cognitive decline but also reduce the negative effects of GV on the nervous system, particularly in specific genotypic and gender groups.

## 7. GV and Infection

### 7.1. Relationship Between GV and Infection in Clinical Studies

GV is strongly associated with infection. In 2018, Shohat et al. studied the effect of GV on postoperative complications following total knee arthroplasty. Their findings indicated that a higher GV was linked to greater risks of periprosthetic joint infection (PJI) and surgical-site infection (SSI). Adjusted analyses signified that for every 10-percentage-point increase in the CV, the length of hospital stay increased by 6.1% and the risks of PJI and SSI by 20% (OR = 1.20) and 14% (OR = 1.14), respectively [112] (Table 5). Lowering the postoperative GV may be vital for decreasing PJI rates in revision hips [113]. Furthermore, a retrospective cohort study examining 13,800 hospitalized surgical patients in New York between 2006 and 2008 reported a distinct link between preoperative and postoperative GV and SSI. Specifically, preoperative GV (OR = 1.11, 95% CI [1.02, 1.21]) and postoperative GV (OR = 1.15, 95% CI [1.07, 1.21] per 10% increase in the CV) were significantly correlated with a higher risk of SSI [114]. In patients undergoing two-stage exchange arthroplasty for PJI, elevated GV has been linked to heightened risks of treatment failure, re-infection, and reoperation [115]. Conversely, a study reported that GV was not associated with PJI, which necessitated revision surgery [116].

In a study involving 1485 patients hospitalized in non-ICU settings with acute infectious diseases, increased GV was linked to elevated risks of bacteremia as well as short- and long-term mortality [117]. Similarly, a high GV has been reported to correlate with an increased risk of ICU-acquired infections and mortality [118]. For patients with T2DM and chronic kidney disease complicated by urinary tract infection, early morning fasting glucose levels, mean blood glucose levels, and their variability are key indicators of severe infection and predictors of renal outcomes [119]. Interestingly, HbA1c variability between primary care visits in patients with T2DM has been associated with more severe infections than average HbA1c levels [120]. After adjusting for the hematopoietic stem cell transplant (HSCT) type, a 4.91-fold (95% CI 1.40–17.24; *p* = 0.013) increased hazard of infection was observed with every doubling of pre-HSCT glucose CV [121]. Similar to studies in adults, GV is associated with pulmonary inflammation in young children with cystic fibrosis [122].

Nonetheless, further well-designed investigations are required to establish the causality of these associations. Although recent studies have reported a robust correlation between GV and various adverse outcomes, such as PJI, SSI, and ICU-acquired infections, the nature of this association remains observational [112,114,115,118].

**Table 5:** A summary of original studies on infection risk associated with GV.

Study	DM Status and Type	GV: Method of Assessment and Parameters	Study Type Included (n)	Outcome	Risk Associated with GV (95% Confidence Interval)	
Goh et al. (2022) [123]	ALL	Visit-to-visit variability of postoperative glucose	Cohort (1983)	Periprosthetic joint infection	Group	OR
					-	1.02 (1.01–1.03) *
Jeon et al. (2012) [114]	ALL	Visit-to-visit variability of preoperative and postoperative glucose	Cohort (13,800)	Surgical-site infection	Group (CV)	HR
					Preoperative glucose	1.11 (1.02–1.21) *
					Postoperative glucose	1.15 (1.07–1.21) *
Subramaniam et al. (2014) [124]	ALL	Visit-to-visit variability of postoperative glucose	Cohort (1461)	Postoperative Complications (including sternal infection and pneumonia)	Group (CV)	OR
					-	1.27 (1.06–1.45) *
Donati et al. (2014) [118]	ALL	Visit-to-visit variability of glucose	Cohort (2782)	ICU-acquired infections	Group	OR
					-	5.04 (1.70–15.00) *
Shohat et al. (2018) [112]	ALL	Visit-to-visit variability of postoperative glucose	Cohort (21,487)	Postoperative Complications	Group (CV)	OR
					Surgical-site infection	1.14 (1.00–1.31) *
					Periprosthetic joint infection	1.20 (1.02–1.41) *
Wang et al. (2020) [115]	ALL	Visit-to-visit variability of glucose	Cohort (665)	Re-infection	Group (CV)	HR
					-	1.31 (1.03–1.68) *
Carey et al. (2024) [120]	T2DM	Visit-to-visit variability of HbA1c	Cohort (411,963)	Hospitalization infections	Group (HbA1c Variability Score)	IRR
					0–20	Reference
					20–50	1.22(1.21–1.24) *
					50–80	1.45(1.43–1.48) *
					≥80	1.67(1.63–1.70) *
Sopfe et al. (2020) [121]	ALL	Visit-to-visit variability of glucose	Cohort (344)	Infection	Group	HR
					-	4.91(1.40–17.24) *

HR, hazard ratio; OR, odds ratio; IRR, incidence rate ratio; GV, glycemic variability; DM, diabetes mellitus; T2DM, type 2 diabetes mellitus; ALL, with or without diabetes; HbA1c, hemoglobin A1c; CV, coefficient of variation; * *p* < 0.05, statistically significant.

### 7.2. Potential Mechanisms of GV in Infection

GV may contribute to microvascular damage, an inflammatory environment, and disruption of normal immune function [29,125,126,127]. GV increases the generation of free radicals. Cells may be unable to adequately enhance their antioxidant defenses, resulting in endothelial dysfunction and oxidative stress during glucose oscillations [29]. When an infection occurs, endothelial cells produce cytokines and regulate the expression of adhesion molecules, enhancing leukocyte extravasation and inflammatory damage. These alterations can trigger tissue injury and worsen outcomes [125].

Hyperglycemia compromises immune function by impairing neutrophil activity, including [126,127]. Moreover, hyperglycemia disrupts the expression of costimulatory molecules, antigen transport, and T-cell activation in specific subsets of dendritic cells, leading to a compromised antiviral adaptive immune response [128]. Therefore, the body’s ability to combat infections is weakened.

GV may augment platelet reactivity and lead to a hypercoagulable state via hyperglycemia. This state may reduce microvascular perfusion when accompanied by thrombus formation, resulting in tissue hypoxia and exacerbating infections. Furthermore, impaired perfusion can impede immune cell migration to infection sites, compromising the host’s infection control ability [80,81].

Overall, GV influences the pathophysiology of infections via its effects on oxidative stress, endothelial dysfunction, inflammatory responses, and immune dysregulation. Effective glycemic control is pivotal for managing infectious diseases.

## 8. Conclusions and Future Perspectives

Owing to advancements in DM management and increased life expectancy, the damaging effects of DM on organs such as the liver, bones, and brain are becoming increasingly evident. Nevertheless, the mechanisms linking DM to these emerging complications are not completely understood. Growing evidence suggests that GV plays a critical role in the development of these complications. This review has integrated research findings on the association between GV and these complications, examining potential mechanisms, including oxidative stress, AGE formation, inflammation, and platelet activation.

Several emerging complications, such as MASLD, share common underlying mechanisms that result in a vicious cycle of hypoglycemia and insulin resistance, exacerbating organ damage. Moreover, complex interactions between complications, such as the indirect effects of MASLD on bone metabolism and fracture risk via increased inflammation and insulin resistance, can augment the effects of GV and complicate disease management.

GV is strongly associated with the onset of nearly all diabetic complications, a relationship that extends beyond diabetic populations. This suggests that GV plays a significant role in the development and progression of these conditions, even in individuals with normal blood glucose levels. Given the substantial health burden and risks linked to these complications, coupled with the critical role of GV, it is crucial for primary healthcare providers to increase their awareness of GV and integrate it as a key focus in glucose management strategies. In addition to routine monitoring of blood glucose and HbA1c levels, a more focused assessment of GV is necessary. For individuals at risk, managing GV is particularly important, as it may serve as an independent risk factor for disease progression. Therefore, establishing optimal GV control ranges is essential, regardless of normal blood glucose levels. Future research should explore the feasibility of targeting GV therapeutically and develop interventions to mitigate its negative impact.

This narrative review has several limitations. First, the lack of a standardized literature search and selection process may limit its reproducibility. Second, the review does not provide further quantitative synthesis of the results. Additionally, potential selection and publication biases may have affected the comprehensiveness of the included studies. Third, many of the studies reviewed are based on Asian populations, which may not fully represent global populations. Future clinical studies involving diverse populations are needed to further explore the role of GV in emerging diabetic complications and its potential as a critical factor in different populations.

## Figures and Tables

**Table 2:** A summary of original studies on the risk of liver disease associated with GV.

Study	DM Status and Type	GV: Method of Assessment and Parameters	Study Type Included (n)	Outcome	Risk Associated with GV (95% Confidence Interval)	
Hong et al. (2021) [53]	No DM	Visit-to-visit variability of FPG	Cohort (57,636)	MASLD	Group (Quartiles of FPG-CV)	OR
					Q1: 4.7 (1.2)	Reference
					Q2: 7.6 (0.7)	1.07 (0.99–1.15)
					Q3: 10.3 (0.9)	1.08 (1.00–1.17) *
					Q4: 15.0 (2.7)	1.15 (1.06–1.24) *
Yoo et al. (2021) [52]	ALL	Visit-to-visit variability of HbA1c	Cohort (21,123)	MASLD	Group (CV of HbA1c)	HR
					NGT	1.01 (0.96–1.07)
					PreDM	1.02 (0.95–1.09)
					DM	1.14 (1.01–1.29) *
Zhou et al. (2022) [51]	T2DM	Visit-to-visit variability of FPG	Cohort (2467)	MASLD	Group (Quartiles of FPG-CV)	OR
					Q1: 4.9 (1.0)	Reference
					Q2: 7.2 (0.6)	1.83 (1.11–3.03) *
					Q3: 9.4 (0.8)	1.53 (0.91–2.56)
					Q4: 19.2 (10.9)	2.80 (1.69–4.64) *

HR, hazard ratio; OR, odds ratio; GV, glycemic variability; DM, diabetes mellitus; ALL, with or without diabetes; No DM, no diabetes; T2DM, type 2 diabetes mellitus; FPG, fasting plasma glucose; MASLD, metabolic dysfunction-associated steatotic liver disease; NGT, normal glucose tolerance; PreDM, Prediabetes; HbA1c, hemoglobin A1c; CV, coefficient of variation; * *p* < 0.05, statistically significant.

## Data Availability

Data sharing is not applicable.
